# From Autism to Eating Disorders and More: The Role of Oxytocin in Neuropsychiatric Disorders

**DOI:** 10.3389/fnins.2015.00497

**Published:** 2016-01-12

**Authors:** Adele Romano, Bianca Tempesta, Maria Vittoria Micioni Di Bonaventura, Silvana Gaetani

**Affiliations:** ^1^Department of Physiology and Pharmacology “Vittorio Erspamer”, Sapienza University of RomeRome, Italy; ^2^Pharmacology Unit, School of Pharmacy, University of CamerinoCamerino, Italy

**Keywords:** oxytocinergic system, autism, eating disorders, anxiety, mood disorders

## Abstract

Oxytocin (oxy) is a pituitary neuropeptide hormone synthesized from the paraventricular and supraoptic nuclei within the hypothalamus. Like other neuropeptides, oxy can modulate a wide range of neurotransmitter and neuromodulator activities. Additionally, through the neurohypophysis, oxy is secreted into the systemic circulation to act as a hormone, thereby influencing several body functions. Oxy plays a pivotal role in parturition, milk let-down and maternal behavior and has been demonstrated to be important in the formation of pair bonding between mother and infants as well as in mating pairs. Furthermore, oxy has been proven to play a key role in the regulation of several behaviors associated with neuropsychiatric disorders, including social interactions, social memory response to social stimuli, decision-making in the context of social interactions, feeding behavior, emotional reactivity, etc. An increasing body of evidence suggests that deregulations of the oxytocinergic system might be involved in the pathophysiology of certain neuropsychiatric disorders such as autism, eating disorders, schizophrenia, mood, and anxiety disorders. The potential use of oxy in these mental health disorders is attracting growing interest since numerous beneficial properties are ascribed to this neuropeptide. The present manuscript will review the existing findings on the role played by oxy in a variety of distinct physiological and behavioral functions (Figure 1) and on its role and impact in different psychiatric disorders. The aim of this review is to highlight the need of further investigations on this target that might contribute to the development of novel more efficacious therapies.

**Figure 1:**
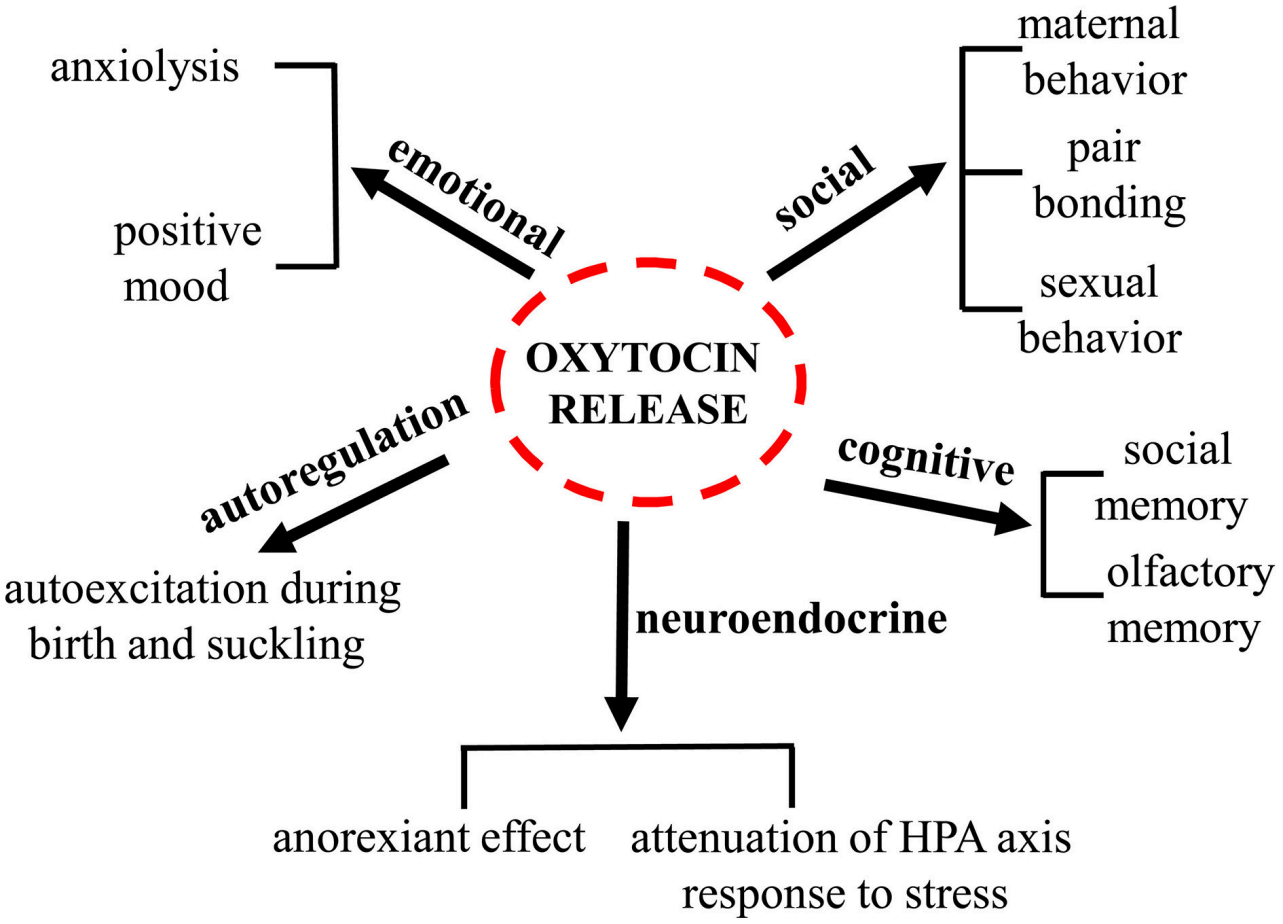
**Oxytocin regulatory control of different and complex processes**.

**Oxytocin regulatory control of different and complex processes**.

## Introduction

### History and structure of oxytocin

The neuropeptide oxytocin (oxy), was discovered in 1906 by Henry Dale, who observed that extracts from human posterior pituitary gland were able to induce uterus contractions in a pregnant cat; the name oxy, derives from the Greek words “ωκνξ τoκoxξ,” meaning “swift birth.”

In 1984, Ivell and Richter elucidated the structure of the oxy gene (Ivell and Richter, 1984), and in 1992 the sequence of the oxy receptor (oxyr) was reported (Kimura et al., 1992). Oxy is a nonapeptides with a disulfide bridge between Cys residues 1 and 6 that creates a six-amino acid cyclic structure and a COOH-terminal α-amidated three-residue tail.

### Synthesis and localization

Oxy is synthesized as a preprohormone precursor protein that includes the oxy carrier protein neurophysin I (Brownstein et al., 1980). Although neurophysin is apparently devoid of any biologically activity, different observations suggests that it might exert a protective role against oxy enzymatic damage and it has been extensively studied for its involvement in the regulation of oxy neurosecretory pathways (Legros and Geenen, 1996; de Bree, 2000). The inactive preprohormone protein is hydrolyzed by a variety of enzymes in small fragments and the last reaction that generates oxy is catalyzed by a peptidylglycine alpha-amidating monooxygenase (Brownstein et al., 1980; Burbach et al., 2001; von Eggelkraut-Gottanka and Beck-Sickinger, 2004). The oxy gene, located on chromosome two in mice, three in rats, and 20 in humans (Dutil et al., 2001), is composed of three exons, each of them encoding for a particular portion of the peptide (Gimpl and Fahrenholz, 2001).

### The oxytocinergic system

#### Hypothalamus-hypophysis

The hypothalamic-neurohypophysial system represents the major oxy neurosecretory system and consists of the paraventricular (PVN) and supraoptic (SON) nuclei (Swanson and Kuypers, 1980; Rhodes et al., 1981) and their axons reaching the neurohypophysis. However, the neurons of PVN and SON project extensively also to other brain areas such as the arcuate nucleus (Arc), the median eminence (ME), the lateral septum (LS) and the medial amygdala nucleus (MeA; Pittman et al., 1981). Within the PVN, two populations of oxy neurons have been identified: “magnocellular” and “parvocellular” neurons. Oxy is mainly synthesized in the magnocellular portions of the PVN and SON (Swaab et al., 1975). Oxytocinergic magnocellular neurons terminate in the posterior lobe of the pituitary gland and also innervate the Arc, the LS, the MeA, and the ME (Pittman et al., 1981). Once activated, magnocellular oxytocinergic neurons, release oxy from the nerve terminals to the posterior pituitary; from here oxy is secreted into the bloodstream, so that it can produce its effect on organs expressing oxyrs located in the rest of the body (Gimpl and Fahrenholz, 2001). The release of oxy from the neurohypophysis into the blood stream is not accompanied by an increase of the peptide at the central level, thus indicating that oxy does not readily cross the blood brain barrier (BBB). In accordance with this observation, peripheral stimulation of oxytocinergic system such as during milk suckling or vaginal dilatation may not change oxy levels in the central nervous system (CSF; Gimpl and Fahrenholz, 2001). Moreover, while stimulation of the PVN evokes oxy release at both central and pheripheral level, electrical stimulation of the rat neurohypophysis only evokes oxy release into the blood (Harris et al., 1981); moreover oxy circulating levels are reduced after hypophysectomy in rats while its concentration increases in the CSF (Dogterom et al., 1977). The oxy half-life is 28 min in the CSF, compared to 1–2 min into the blood; in the CSF, oxy is normally present at concentrations of 10–50 pM, slightly higher than those in plasma (Jones and Robinson, 1982; Meyer et al., 1987). Moreover, oxy release has been shown to occur not only at the axonal levels but also locally from dendrites in both the PVN and SON (Neumann et al., 1996).

Furthermore, oxy can act as an intrinsic self neuromodulator; oxy release within the SON is pivotal for the coordinated depolarization of oxy neurons during lactation and for the positive feedback mechanism mediated by oxyrs on its own release during parturition (Neumann et al., 1996).

The parvocellular neurons, that are smaller than magnocellular neurons, are located in the dorsal-caudal portion of the PVN and terminate principally to the nucleus of solitary tract (NST), the dorsal motor nucleus of the vagus (DMNV), the rostral ventrolateral medulla, and the sympathetic centers in the spinal cord (Amico et al., 1990; Rinaman, 1998; Tóth et al., 1999).

Finally, it has been proposed that oxy may participate in the physiological regulation of the adenohypophysial hormones prolactin adrenocorticotropic hormone (Page et al., 1990), and gonadotropins (Robinson and Evans, 1990). This hypothesis is supported by evidence demonstrating that hypothalamic oxytocinergic fibers reach also the anterior part of the pituitary gland via the hypophyseal portal system (Amar and Weiss, 2003).

#### Oxytocin receptor

The oxy receptor (oxyr) is a 389 aminoacid polypeptide belonging to the G-protein coupled receptor family with seven transmembrane domains. Specifically this receptor is coupled to a Gq/11α protein that stimulates the activity of phospholipase C. This leads to the generation of second messengers, release of Ca^2+^ from the intracellular storages, and activation of protein kinase type C. These two conditions can trigger several cellular events such as the contraction of smooth cells, cellular excitation, and modification of gene expressions (Gimpl and Fahrenholz, 2001).

The oxyr has been identified not only in the brain but also in several peripheral organs. In the rat CNS, oxyrs are present in several regions, including the olfactory system, cortex, thalamus, basal ganglia, ventromedial region of the hypothalamus, bed nucleus of the stria terminalis, central amygdala, ventral subiculum, hippocampus, nucleus accumbens, brain stem, and spinal cord (Gimpl and Fahrenholz, 2001). Interestingly, their expression density changes according to the age (Gimpl and Fahrenholz, 2001).

In human brain oxyrs are not present in the olfactory bulbs, entorhinal cortex nor in the amygdala or hippocampus but they are mainly expressed in the pars compacta of substantia nigra and globus pallidus, as well as in the anterior cingulate and medial insula (Gimpl and Fahrenholz, 2001). In peripheral organs oxyrs are expressed in the uterus, mammary gland, ovary, kidney, heart, bone, and endothelial cells (Gimpl and Fahrenholz, 2001). Through the activation of oxyrs in the bone tissue oxy was shown to be implicated in bone growth and in the remodeling of bone structures (Breuil et al., 2011).

#### Neuromodulatory control exerted by oxytocin

Similarly to other neuropeptides, oxy is able to influence neurotransmission within the nervous system in a manner that is different from other classical neurotransmitters such as GABA, serotonin and dopamine (reviewed by Ludwig and Leng, 2006). The first main difference between the action of neuropeptidergic and classical neurotransmitters is linked to their very different turnover: neuropeptides show a much longer half-life (about 20 min) than neurotransmitters (about 5 ms), due to their much slower degradation (Mens et al., 1983). As a consequence, neuropeptides can produce their effects in the CNS at longer distances and in broader regions of diffusion (Landgraf and Neumann, 2004; Ludwig and Leng, 2006).

The second main difference concerns the modality of their release, being neuropeptides stored in core-dense vesicles that are released not only at the axonal endings but also at the somatodendritic levels and are much larger than synaptic vesicles that store and release classical neurotransmitters at the synaptic terminals (Morris and Pow, 1991). Both these two aspects make the neuromodulatory actions of neuropeptides much broader and less spatial/temporal specific with respect to classical neurotransmitters. However, at the same time, neuropeptidergic neurons form also synaptic connections with other neurons and, therefore, neuropeptides are able to play also a more specific action at their axon terminal that is more similar to the function of other classical neurotransmitters. The oxytocinergic system shares these common aspects with many other neuropeptides.

How oxy can actually affect neurotransmission in different brains areas and the effects that this modulation can produce on behaviors and neuronal function has started to be elucidated recently.

Oxy is able to rapidly change brain state to modulate synaptic plasticity in specific areas. These includes the mouse auditory cortex, which has been recently demonstrated (Marlin et al., 2015), or the hippocampus (Mühlethaler et al., 1984; Zaninetti and Raggenbass, 2000; Owen et al., 2013), or the nucleus accumbens (Dölen et al., 2013). In particular it has been emerging that in these areas oxy neuromodulation acts sharpening neuronal responses to increase the salience of specific stimuli. This action is the result of a dual action: at the signal generation that is increased and at the background noise that is dampened. For example, Marlin and colleagues have studied the role of oxy in the mouse maternal behavior of pup retrieval and demonstrated that oxy is able to regulate the neuronal responses induced by pup calls in the auditory cortex of the mothers. This action is not symmetrical in both hemispheres and leads to a lateralization of the response with a greater stimulation of the left auditory cortex, where a higher density of oxyr is observed mostly on inhibitory interneurons expressing parvalbumin and somatostatin. The activation of these receptors by oxy released from projections of PVN neurons seems to be required mainly to trigger, rather than to maintain, the retrieval behavior and it is able to produce a specific equilibrium of the degree and timing of inhibitory signal with excitatory signal, which produces and increase of the salience of pup calls.

A similar mechanism was demonstrated also in the hippocampus. At this level oxyr are mostly expressed in the soma and dendrites of GABAergic interneurons, whose firing is increased by their activation. Interneuron firing, in turn, suppresses the activity of pyramidal neurons (Mühlethaler et al., 1984; Zaninetti and Raggenbass, 2000). Owen et al. (2013) showed how this positive effect of oxy on the firing of fast-spiking interneurons is able to enhance the signaling to cortical structures while concurrently lowering the background noise. In fact, the increased firing of fast-spiking interneurons is able to suppress the spontaneous firing of pyramidal hippocampal neurons. At the same time, a use-dependent depression develops at the synapse between the fast-spiking interneuron and the pyramidal cell that leads to the enhancement of spike throughput. This circuitry, which can be activated by any manipulation able to stimulate fast-spiking interneurons, leads to a significant enhancement of the fidelity and timing of spike transmission through the network.

In the nucleus accumbens oxy was demonstrated to act causing a presynaptic long term depression (LTD) of excitatory inputs that plays a key role in the positive reinforcing properties of social interaction in rodents (Dölen et al., 2013). In particular, Dölen and collaborators demonstrated the expression of oxyr on the axon terminals of serotonergic neurons arising from the dorsal raphe nucleus. The activation of oxyr at these terminals is able to induce a presynaptic form of LTD in medium spiny neurons through the involvement of the subtype 5HT1B of serotonergic receptors and such mechanism plays a necessary role for social reward in mice. Recent findings from the Piomelli's laboratory demonstrated that this mechanism of synaptic plasticity triggered by oxy in the nucleus accumbens involves also another important neural component of the circuitry: the endocannabinoid system. In fact, the activation of oxyr is able to trigger the synthesis and release of anandamide in the nucleus accumbens that, in turn, activates local CB1 receptors (Wei et al., 2015). The involvement of these receptors appears necessary in mediating the rewarding properties of social interaction. This observation suggests the hypothesis that CB1 activation in the nucleus accumbens might participate in the oxy-triggered synaptic plasticity that mediates oxy effects in this behavioral context, as the activation of CB1 receptors has been demonstrated to induce presynaptic LTD in other brain stuctures (Robbe et al., 2002; Gerdeman and Lovinger, 2003).

These mechanisms of oxy action in different brain structures provide insight into how a diffusely distributed neuromodulatory signal can specifically influence the performance of complex networks in a manner that ensure synapse specificity and millisecond definition.

### Physiological role of oxytocin

#### Oxytocin in the lactation and parturition

The oxytocinergic system plays a crucial role in the induction of labor, due to its uterotonic action exerted on the endometrium smooth muscle. Therefore, selective oxy receptor antagonist may be useful for the prevention of premature delivery and dysmenorrhea (Williams et al., 1998; Blanks and Thornton, 2003; Leng et al., 2005).

During parturition, the mechanical stimulation of the uterus provokes, via the vagal and pelvic nerves, the activation of the NST via A2 adrenergic cells (Russell et al., 2003) and the ventromedial medulla, which, in turn, projects to magnocellular neurons. During lactation, oxy induces milk ejection, acting on myoepithelial cells of the mammillary glands (Blanks and Thornton, 2003). A reflex response to the stimulation of the nipple by suckling is able to activate oxy secretion not only from the axons reaching the neurohypophysis, but also from the soma and dendrites of oxy neurons in the SON, where oxy acts in a paracrine manner to trigger and maintain a synchronized bursting (Richard et al., 1997; Ludwig, 1998). This mechanism occurs in concert with other important factors that contribute to milk ejection, such as the sight, smell, and sound of the newborn (Leng et al., 2005).

#### Oxytocin and social behavior

Oxy seems to be implicated in several and complex social behaviors in a range of mammals (Carter, 1992; Bosch and Neumann, 2012). One of the most studied aspects is the regulation of maternal behavior (Bosch and Neumann, 2012). In particular, oxy promotes maternal care in lactating rats and plays a key role in bond formation between mother and offspring (reviewed by Ross and Young, 2009). When central oxy production is blocked or after the central administration of an oxyr antagonist the maternal bond formation is impaired (Ross and Young, 2009). In addition, oxy is also critical for the formation of the bond from the infant to his mother, as suggested by the experimental observation that mouse pups lacking oxy gene fail to prefer their biological mother over a foster ones (Ross and Young, 2009).

Oxy is also involved in the mechanisms regulating the development of adult-adult pair; in fact, oxy plays a role in the partner preference of monogamous prairie voles. In particular, in female prairie voles central administration of oxy induces pair-bonding without prior mating (Williams et al., 1994) and this behavior is blocked by the treatment with oxyr antagonist given before mating (Insel and Hulihan, 1995). The identification of conspecifics is a crucial requirement for the formation of pair bonds (Insel and Fernald, 2004); oxy takes part to the processes that regulate social memory, in terms of social learning and social recognition (Benelli et al., 1995; Engelmann et al., 1998) and the pretreatment with an oxyr antagonist prevents this ability (Benelli et al., 1995; Engelmann et al., 1998; Lukas et al., 2013).

Interestingly, the administration of a low dose of oxy, within the amygdala, re-establish normal social behavior in oxy null mice (Ferguson et al., 2002). The observation that this effect occurs when oxy administration precedes the first exposure to a conspecific during the test of social recognition suggests that the oxytocinergic system in mice is crucial for the memory processes associated with the acquisition of the social information rather than the consolidation (Ferguson et al., 2002). In accordance with such hypothesis, the central administration of an oxyr antagonist in the medial part of the amygdala results in impairment of recognition of conspecifics encountered previously (Ferguson et al., 2002). The effects of oxy on social behavior are complex and may be dependent on the context in which they occur. In fact, oxy seems to promote sociality when social cues in the environment are interpreted as “safe” and oxy may induce defensive behavior when the social cues are interpreted as “unsafe” (Bartz et al., 2011; Olff et al., 2013). This latter effect is also present when oxy is administered to subjects with an adverse early history (Bhandari et al., 2014).

Finally, it is well known that “mind reading” is an essential basis of human social interaction and the role that oxy plays in the formation of social bonds is also related to its role of promoting the understanding of the mental state of individuals from the interpretation of their facial cues (Domes et al., 2007).

#### Oxytocin and stress

Oxy release from the pituitary gland occurs also in response to different stressful stimuli, such as conditioned fear, pain, electric footshock, exposure to novel environments, restraint stress, etc. The stimulation of oxy release under these circumstances is mediated, at least in part, by noradrenergic neurons containing prolactin-releasing peptide in the NST (Onaka, 2004). Also in humans acute psychologically stressful stimuli have been shown to increase plasma oxy concentrations (Sanders et al., 1990; Pierrehumbert et al., 2010).

A large body of evidence indicates that oxy displays potent anxiolytic properties and dampens the neuroendocrine response of the hypothalamic-pituitary-adrenal (HPA) axis to stress (Viero et al., 2010). Intriguingly Dabrowska and collaborators (Dabrowska et al., 2011) have shown the presence of a potential reciprocal interaction between the hypothalamic oxytocinergic system and the corticotrophin-releasing factor (CRF) system. The authors demonstrated that CRF neurons of both PVN and bed nucleus of stria terminalis would be highly responsive to local oxy release, leading to the onset of an inhibitory circuit, which could reduce the HPA axis activation in response to stress.

#### Oxytocin and sexual behavior

Oxy has been strongly linked to the stimulation of sexual behavior and it is released in response to sexual interactions in rats (Carter, 1992). Also in humans, both man and women, oxy plasma levels are increased during orgasm and sexual arousal (Carmichael et al., 1987; Carter, 1992). In male rats oxy facilitates copulation, when injected into the medial preoptic area and this effect is abolished by the pre-administration of an oxyr antagonist (Gil et al., 2011). Moreover, oxy injection in the ventral tegmental area (VTA) induces penile erection by interacting with the dopaminergic system (Melis et al., 2007; Baskerville et al., 2009). Interestingly, intranasal oxy administration evokes in women a marked increase of sexual desire, which is associated with vaginal transudate (Anderson-Hunt and Dennerstein 1994, 1995).

Finally, oxy levels fluctuate throughout the menstrual cycle in fertile women not using oral contraceptives; in particular during the luteal phase oxy plasma levels are lower compared to those observed in the ovulatory phase (Altemus et al., 2001; Salonia et al., 2005). Moreover, during the ovulation, the endogenous oxy activity is suppressed (Evans et al., 2003).

#### Oxytocin and cardiovascular control

Both magnocellular and parvocellular PVN neurons are involved in the autonomic control of the heart and vessels and a role of central oxy with respect to cardiovascular control has been established (Coote, 2005) demonstrating also the expression of oxyrs in cardiomyocytes and in the wall of large blood vessels (Gutkowska et al., 1997; Jankowski et al., 1998, 2000); the content of oxyrs in the heart is lower than in the uterus, but the size of the receptor is similar (Gutkowska et al., 1997; Cicutti et al., 1999). The rat heart is a site of oxy synthesis and release; in particular oxy was detected in all four chambers with the highest concentration in the right atrium. The heart oxy is structurally identical, to the oxy found in the hypothalamus (Jankowski et al., 1998).

The oxytocinergic system in rat heart is physiologically relevant for a number of reasons:

1. oxy is a cardiomiogenic factor. In fact oxy induces the differentiation of the so called “side population” (SP) progenitor cells into beating cardiomyocytes (Oyama et al., 2007); moreover oxy treatment of the progenitor cells expressing the stem cell antigen-1 (Sca-1) induces the gene expression of cardiac transcription factors, contractile proteins, and stimulates spontaneous beating (Matsuura et al., 2004).
2. oxy exerts a cardioprotective action by stimulating the heart release of the atrial natriuretic peptide (ANP; Favaretto et al., 1997; Gutkowska et al., 1997) a well established cardioprotective agent (Kasama et al., 2008).
3. oxy produces a negative cronotropic effect which is protective on ischemia-reperfusion-induced myocardial damage. In particular, Ondrejcakova and his collaborators showed that perfusion with oxy before ischemia resulted in a reduction of the infarct size (Ondrejcakova et al., 2009). Both central and peripheral administration of oxy induce a long-term decrease of blood pressure in rats (Petersson et al., 1996) and cause a concentration-dependent reduction in both heart rate and force of contraction of isolated atria (Favaretto et al., 1997). Interestingly, changes in brain oxy content were found in spontaneously hypertensive rats (SHRSP). In particular, oxy content was markedly reduced in the hypothalamus, the brain stem and spinal cord of SHRSP, compared to age-matched normotensive rats (Gaida et al., 1985) and SHRSP rats were found to express lower levels of oxy mRNA in the SON and PVN (van Tol et al., 1988).

#### Oxytocin and analgesia

The analgesic properties of oxy seem to derive mostly from its interaction with the central endogenous opioid system (Gu and Yu, 2007; Han and Yu, 2009).

From the hypothalamus, oxy is transported not only to the pituitary gland but also to other brain areas involved in the nociceptive signaling such as the amygdala (Gimpl and Fahrenholz, 2001; Crock et al., 2012) and to the spinal cord in the dorsal horn (Zimmerman et al., 1984), where it modulates pain perception (Yang et al., 2007a) Several behavioral studies in rodents demonstrated that intrathecal administered oxy may exert antinociceptive effects in a dose-dependent manner (Lundeberg et al., 1993; Xu and Wiesenfeld-Hallin, 1994; Yang et al., 2007a). Similarly, oxy was found to induce a dose-dependent increase in the hindpaw withdrawal latency to noxious thermal and mechanical stimulation in rats, when injected into the nucleus accumbens or in the central amygdala, whereas the administration of the oxyr antagonist atosiban in this latter area was able to block oxy antinociceptive effect (Gu and Yu, 2007; Han and Yu, 2009). Intraperitoneal administration of oxy was able to decrease the licking/biting response of the formalin injected pow in a model of tonic continous pain in mice and such effects was shown to involve opioid receptors (both κ and δ) and voltage-gated calcium channels (Reeta et al., 2006). Moreover, endogenous oxy was shown to attenuate the vocalization induced by electrical whisker pad stimulation in decerebrated newborns by inducing a reduction of the depolarizing action of GABA on nociceptive neurons; an effects that was blocked by the administration of an oxyr antagonist (Mazzuca et al., 2011).

A variety of non noxious stimuli stimulate the oxytocinergic system; thermal stimulation (40°C), vibration (100 Hz), and electro-acupuncture (2 Hz) induce a significant increase of oxy levels in plasma and/or in CSF of rats (Uvnäs-Moberg et al., 1993). On this regard Yang and his collaborators demonstrated that central oxy administration enhances acupuncture analgesia, while central administration of anti-oxy serum weakened acupuncture analgesia in a dose-dependent manner (Yang et al., 2007b). Oxy was also shown to exhibit antinociceptive effects in neuropathic animals where it reduced post-discharge produced by electrical stimulation (Condés-Lara et al., 2005). A fewer set of data is available in support of the analgesic role of oxy in humans. One study indicated that low oxy levels are significantly associated with ratings of greater pain, stress, and depression in patients affected by fibromyalgia (Anderberg and Uvnäs-Moberg, 2000). In another clinical study intrathecal oxy acute administration was able to relieve from low back pain (Yang, 1976), through a mechanism likely involving the endogenous opiate system, as suggested by the effects of naloxone pre-administration in the prevention of such effect.

## Oxytocin and neuropsychiatric disorders

### Oxytocin and autism

Autism spectrum disorders (ASD) refer to a group of complex and prevalent developmental impairments characterized by deficits in social interaction, verbal skills, repetitive behavior, and reduced range of interests and activities (Muhle et al., 2004). As already described in previous paragraphs, oxy is implicated in social recognition, attachment, and stereotyped behaviors (Insel et al., 1999; Ferguson et al., 2000; Takayanagi et al., 2005); moreover oxy has a recognized role in anxiety (Labuschagne et al., 2010; Amico et al., 2014), which is often a comorbid feature of autism (South et al., 2011; Vannucchi et al., 2014).

Deficits in oxy signaling mechanism have been proposed to contribute to the onset of several behavioral abnormalities of ASD (Insel et al., 1999; Insel and Young, 2001). Given the potential link between oxy functions and core deficits in ASD, oxy has received increasing attention in the last years as a potential therapeutic target for these disorders.

Human studies have demonstrated that oxy promotes retention of social information and reduced repetitive behaviors (Hollander et al., 2003, 2007) in individuals with ASD. Moreover, studies performed on children and adolescents with ASD subjected to intranasal oxy administration over a period of 2–6-month suggest that oxy improves social communication in these individuals (Kosaka et al., 2012; Tachibana et al., 2013).

A direct involvement of oxy in autism has been suggested already in 1998 by Modahl et al. (1998), who reported decreased plasma oxy levels in autistic children, as compared with age-matched healthy controls. In this study, a decrease in oxy levels correlated to a low development of social and communication skills.

Interestingly, it has been shown that individuals with autism display a defect in processing of the peptide oxy; in these subjects the decreased plasma oxy levels were associated with an increase of oxy-extended peptide inactive form (Green et al., 2001). This inactive peptide form, which is normally detected during the fetal life, is present in high quantity in the blood of autistic children (Green et al., 2001), thus possibly interfering with the functioning of the oxytocinergic system.

However, oxy levels did not correlate with impairments in social interaction, communication, or stereotyped behavior in adult subjects suffering of ASD, as measured by the Autism Diagnostic Interview-Revised (ADI-R) (Lord et al., 1994). On the same line, Jansen et al. (2006) found that ASD adults showed increased basal oxy plasma levels. Based on these observations, it seems that the oxytocinergic system might become dysfunctional during different developmental stages (adults vs. children) over the lifespan of individuals with ASD, but further studies are necessary to confirm these findings.

Several large-scale studies have demonstrated the association between genetic vulnerability on the gene encoding oxyr and ASD. Specific gene polymorphisms for oxyr gene were associated to autism in the Han Chinese population (Wu et al., 2005). On the same line, Jacob et al. (2007) revealed a similar finding in autistic Caucasian children and adolescents. These findings were extended by Lerer et al. (2008), who demonstrated not only a significant association between SNPs and ASD but they also reported an association between oxyr variants, intelligence quotient and scores of the Vineland Adaptive Behavioral Scales (VABS), an effective assessment tool to evaluate the social abilities of an individual. Moreover, Yrigollen et al. (2008) found a significant association of both the oxy gene (two SNPs) and oxyr (three SNPs) and autism diagnosis as well as Campbell et al. (2011) and his collaborators reported an association of oxyr genetic polymorphisms with social communication dysfunction in ASD subjects.

Conversely, Tansey et al. (2010) failed to demonstrate an association between 18 single nucleotide polymorphisms (SNPs) in oxyr gene and ASD, although, the authors did not evaluate the SNP mostly involved (rs2254298) in the studies performed before.

Interestingly, oxyr gene expression may be affected by epigenetic modifications, as suggested by the observation of a significant association between methylation of oxyr and decreased oxyr mRNA levels in the temporal cortex tissue collected post mortem from individuals affected by autism (Gregory et al., 2009).

The evidences reported above suggest that there is still lack of consistency among different studies and probably this is due to the heterogeneous nature of ASD and to the enormous quantity of genes to consider.

A large number of studies have investigated the impact of exogenous oxy administration in individuals with ASD. Oxy intravenous administration reduced repetitive behaviors in adults with ASD compared to placebo (Hollander et al., 2003). In a second experiment (Hollander et al., 2007), adult subjects diagnosed with autism were treated with intravenous oxy and were subjected to a task in which they had to identify the mood of the person who held the speech which pronounced four sentences of four emotional intonations (happy, indifferent, angry, sad). All subjects treated with oxy improved the comprehension of affective speech from pre- to post-infusion and the authors interpreted this result as oxy increasing retention of social cognition.

Intranasal oxy administration has been demonstrated to improve the performance of both adolescent (Guastella et al., 2010) and adult (Domes et al., 2007) males with ASD on the so-called “Reading the Mind in the Eyes Task” (RMET) that provides the identification of emotions on the basis on the observation of the eyes of a face. Moreover, intranasal oxy administration increased total gaze time spent on face regions by increased fixation time on the eye region displayed by adults with ASD (Andari et al., 2010). Finally, long-term daily intranasal oxy administration improved social behaviors in both male and females with ASD (Anagnostou et al., 2012; Kosaka et al., 2012).

In accordance with this large set of clinical data, subcronically administered oxy was able to improve the social deficits and the repetitive behavior that can be observed in two inbred mouse strains (Teng et al., 2013), which exhibit some of the behavioral abnormalities of subjects suffering from ASD, such as the BALB/cByJ mice (Moy et al., 2007) and the C58/J mice (Ryan et al., 2009). Another preclinical evidence in support of the role of oxy in ASD derives from studies conducted on mice lacking the gene that codifies for the cell-adhesion molecule contactin-associated protein-like 2 (Cntnap2), which in humans is associated to a syndrome characterized by cortical dysplasia, focal epilepsy and, in most cases, ASD. These mice have a decreased number of oxy neurons in the PVN and a general reduction of oxy levels and show deficits of social behavior that an acute intraperitoneal treatment with oxy is able to restore (Peñagarikano et al., 2015).

The distribution of oxyrs in specific brain areas involved in social behavior, overlap with μ-opioid receptors and this observation suggest the existence of an interaction between the two systems that might play a role in ASD. Gigliucci and collaborators, contributed to the understanding of the role of oxy in ASD by testing oxy effects in mice lacking μ-opioid receptors, which are considered an experimental model of autism since they display abnormal social interaction, increased self-grooming, stereotyped behavior, and a general a of sociability (Gigliucci et al., 2014). These mice display an increase in oxyrs expression in the nucleus accumbens, medial and central amygdala, and in the medial anterior olfactory nucleus; intranasal oxy administration to these mice was able to rescue the social impairments observed in different behavioral tests (Gigliucci et al., 2014).

Importantly, it is known that there is a window of time in post-natal life of the rodent, during which there is a peak in the concentration of oxyr in the neocortex (Hammock and Levitt, 2013) and administration of oxy in this age can be responsible of several behaviors that the individual can develop later in adulthood. On this line it has been reported that when oxy is administered daily in the first postnatal week can prevent deficits in social behavior and learning abilities in mice deficient for the melanoma antigen family L2 (Magel2), a gene which has been found mutated in patients with autism (Meziane et al., 2014).

Recent advances in understanding possible neural systems implicated in the etiopatogenesis of autism have highlighted that some forms of autism might be caused by an imbalance between excitation and inhibition at various neuronal systems. It has been reported that both autistic human subjects and animal models of autism display dysfunction in GABA signaling (Rubenstein and Merzenich, 2003; Gogolla et al., 2009; Blatt and Fatemi, 2011). Interestingly, GABA which in adults has an inhibitory role, during early development plays an excitatory action (Ben-Ari et al., 2007). The shift from excitatory to inhibitory, occurs at birth, and has been shown that oxy plays a key role in this process, causing a reduction in the intracellular concentration of Cl^−^ (Tyzio et al., 2006) and this effect disappears in CA3 hippocampal neurons of two mouse model of autism: mouse *in utero* exposed to valproate and mice carrying the fragile x mutation (Tyzio et al., 2014). In the same study, the authors also reported that prenatal treatment with the oxy receptor antagonist SSR126768A in naïve animals induces alterations similar to those observed in both mouse models of autism (Tyzio et al., 2014).

In summary, all these evidences point toward oxy potential as an agent to improve social cognition, functioning and repetitive behavior in ASD, although the literature still very tentative due to methodological constraints.

### Oxytocin and eating disorders

Several lines of evidence have established a link between oxy signaling and food intake and in the last years this peptide has gained attention for its effects in the treatment of obesity (Kublaoui et al., 2008; Maejima et al., 2009, 2011; Deblon et al., 2011; Zhang and Cai, 2011; Zhang et al., 2011; Morton et al., 2012). Clinical investigations list oxy in a high number of studies on caloric intake, gastric emptying, and obesity (Blevins and Ho, 2013).

Meal-related stimuli such as the intake of food (Johnstone et al., 2006), the release of the satiety signal cholecystokinin (Olson et al., 1992;), gastric distension (Renaud et al., 1987; Nelson et al., 1998), or stimulation of gastric vagal afferents (Ueta et al., 2000; Tang et al., 2006) are associated with activation of oxy neurons within the PVN and SON, release of oxy into the bloodstream, and activation of hindbrain areas that regulate meal size.

Oxy- and oxyr knock out mice develop late-onset obesity (Takayanagi et al., 2008; Camerino, 2009) and both systemic (Arletti et al., 1989, 1990) and central oxy administration decrease food intake; moreover the pre-treatment with an oxy receptor antagonist blocks this effect (Arletti et al., 1989, 1990; Olson et al., 1991). Oxy treatment has been evaluated in several animal models of obesity. In particular, central oxy chronic treatment induces a dose-dependent decrease in body weight gain, stimulates lipolysis and fatty acid β-oxidation, reduces glucose intolerance, and insulin resistance in diet-induced obese (DIO) rats (Deblon et al., 2011; Morton et al., 2012). Similarly, subcutaneous daily oxy administration reduces food intake, body weight, ameliorates fatty liver, and glucose intolerance in DIO mice (Maejima et al., 2011). In accordance with this observation, a very recent study demonstrated that a 4-week chronic oxy treatment reduces body weight in DIO rhesus monkeys by decreasing food intake and increasing energy expenditure and lipolysis (Blevins et al., 2015).

Oxy antiobesity treatment has proved valid also in animal models that exhibit an altered leptin signaling, in fact oxy induces anorexia in leptin-resistant Zucker-fatty rats (Maejima et al., 2009), causes a dose-dependently reduction in food intake and body weight gain in ob/ob animals (Altirriba et al., 2014) and suppresses food intake by activating vagal afferent neurons in leptin-resistant db/db mice (Iwasaki et al., 2015). Specifically, oxy decreases food intake by reducing meal size and increasing the latency to the first meal, while oxy antagonists stimulate food intake by increasing meal size (Arletti et al., 1990). Moreover, oxyr null mice show an increase in meal size during the dark cycle (Takayanagi et al., 2008). Interestingly, it has been shown that oxy injections into the dorsal vagal complex inhibits gastric motility in rats (Rogers and Hermann, 1987) and this is in line with the findings that oxy excites neurons of both NST and DMNV, which respond to gastric distension (McCann and Rogers, 1990).

Consistently with the presence of oxyrs along the gastrointestinal tract (Qin et al., 2009), it has been shown that systemic oxy reduces gastric empting in laboratory rats and this effect is blocked by the pre-treatment with an oxyr antagonist (Wu et al., 2003, 2008). Moreover, it has been suggested that the anorexiant effect of oxy might involve in part the inhibition of reward circuits: in fact, Carson and collaborators demonstrated that systemic oxy reduces methamphetamine-induced activity in reward related brain areas. Furthermore, the same group demonstrated that systemic oxy reduces the motivation to consume methamphetamine and reduces the methamphetamine induced hyperactivity in mice (Carson et al., 2010a,b). These results support the potential use of oxy as a beneficial treatment for addictive disorders. Interestingly, in a recent research (Ott et al., 2013) Ott and collaborators examined the food intake and reward-driven snack intake in humans subjected to intranasal oxy administration. The authors found that oxy markedly reduced the intake of chocolate cookies, further supporting a pivotal contribution of oxytocinergic system in the regulation of reward-related eating behavior. In addition to inhibit food intake, both central and pheripheral oxy administrations reduce body weight by increasing energy expenditure (Morton et al., 2012) and lipolysis (Muchmore et al., 1981). In contrast, oxy antagonist increases body weight gain (Zhang and Cai, 2011) and mice genetically lacking oxy or oxyr develop obesity during adulthood (Camerino, 2009). In accordance with these observations, mice modified genetically to have a reduced PVN oxy signaling, such as single-minded 1 gene (SIM1) haploinsufficient mice (Xi et al., 2012) or synaptotagmin 4 (SYT4) null mice (Zhang et al., 2011), are characterized by a reduction in energy expenditure, hyperphagia and obesity. The excessive body weight in SIM1 haploinsufficient mice can be restored by oxy treatment (Kublaoui et al., 2008). Likewise, humans with mutations of SIM1 show severe eating disorders (Holder et al., 2000; Swarbrick et al., 2011). Whether oxy treatment would be able to control body weight gain in this subject remained unexplored.

Although research on the implication of the oxytocinergic system in anorexia nervosa (AN) are at the beginning and all those existing are based on small sample sizes, it has been suggested that the complex array of neuroendocrine disturbances in AN involves the oxytocinergic system. Demitrack et al. (1990) reported that women affected by restricting anorexia show oxy CSF levels significantly lower than the levels observed in control subjects. Moreover, women affected by AN display a decreased overnight secretion of oxy compared to healthy controls and this is associated with a decrease in bone mineral density and body fat (Lawson et al., 2011). Furthermore, both bulimic and anorectic patients present lower serum activity of the prolyl-endopeptidase, an enzyme implicated in the cleavage of several active neuropeptides, such as oxy (Maes et al., 2001). Finally, results from two pilot studies revealed changes on eating-related indices, following intranasal administration of oxy in AN subjects, compared to placebo. In particular in one study, the researchers observed a reduction in eating disorder and in the other study they found that oxy significantly reduced selective attention toward anxiety laden eating stimuli (Maguire et al., 2013).

#### Prader-willi syndrome

The Prader Willi syndrome (PWS) is a genetic disorder characterized by hypotonia, developmental disability, hypogonadism and, importantly, gross body weight gain and insatiable hunger due to impaired perception of satiety.

In animal research, it has been shown that mice lacking one of the members of the melanoma antigen gene (MAGE) family, called MAGED1 display an increase in daily food intake accompanied by a reduction in mature hypothalamic oxy levels; moreover they develop adult-onset obesity and reduced activity (Dombret et al., 2012). MAGED1 together with MAGEL2 and Necdin are genes critically involved in PWS (reviewed by Francis et al., 2014). It has been shown that MAGEL2 play a crucial role in suckling, thus MAGEL2-deficient mice had a high mortality in the neonatal life, which is due to their suckling defects. Interestingly, these mutant mice display a reduction in oxy hypothalamic levels and a single injection of oxy soon after birth, rescued the phenotype of Magel2 mutant pups, allowing them to survive (Schaller et al., 2010).

Finally Necdin mutant mice showed a reduced number of oxy producing neurons (Muscatelli et al., 2000) and presented respiratory problems that evoke those observed in PWS patients (Wharton and Bresnan, 1989).

In PWS subjected, intranasal oxy administration increased trust in others and decrease sadness and disruptive behavior (Tauber et al., 2011). In addition, results from a postmortem study of PWS subjects, reported a 42% reduction of oxy-expressing neurons in the PVN and a smaller oxy cells volumes, as compared to healthy controls (Swaab et al., 1995). Moreover, PWS subjects present increased oxy CSF levels similarly to obsessive compulsive disorders (OCD) patients (reviewed by Marazziti and Catena Dell'osso, 2008). Interestingly, the prevalence of OCD in PWS is high (Dykens et al., 1996), suggesting that this two pathologies might share common abnormalities at the level of the oxytocinergic system.

### Oxytocin and other psychiatric disorders

Given to its effect on cognition, memory, and social functioning, oxy has been studied in the pathophysiology of a wide range of psychiatric disorders including schizophrenia, mood, anxiety, and obsessive compulsive-disorders. In the present paragraph we report both preclinical and clinical findings obtained in studies that evaluated the activity of the oxytocinergic system in these disorders.

Both preclinical and clinical literature suggests that oxy has a role in schizophrenia. In mice, for example, it has been demonstrated that the oxytocinergic system is affected and that oxy may explicate its potential antipsychotic effect through the inhibition of the mesolimbic dopaminergic circuit (Macdonald and Feifel, 2012). Moreover, a positive correlation between plasma/CSF oxy levels and schizophrenia has been reported. In particular, it has been found an increase of CSF oxy levels in adult males with paranoid schizophrenia and increased plasma oxy level in schizophrenic patients particularly in those taking neuroleptics (Beckmann et al., 1985).

Interestingly, in a very recent study, it has been reported that the higher plasma oxy levels in schizophrenic patients might be due to a compensatory response to a lower sensitivity of the oxyr to circulating levels of the hormone (Strauss et al., 2015). Similar findings were obtained also from the measure of the levels of human neurophysin II (hNPII). Interestingly CSF levels (Linkowski et al., 1984) and serum level (Legros et al., 1992) of hNPII were increased in individuals with schizophrenia compared to healthy volunteers. In contrast to the evidences reported above, no differences in oxy concentrations in the CSF (Glovinsky et al., 1994; Sasayama et al., 2012) or plasma (Goldman et al., 2008) were found between patients with schizophrenia and healthy controls. We think that inconsistencies in oxy levels among studies may reflect differences in evaluating peripheral vs. cerebrospinal fluid levels, sample-related differences, sex, age, race, and differences in disease chronicity.

Interestingly, genetic studies revealed that two different oxyr SNPs were associated with schizophrenia (Montag et al., 2013) and Souza et al. (2010) evaluated the association between both oxy and oxyr variants with symptom severity and the response to clozapine in individuals with schizophrenia, since it has been demonstrated that clozapine enhances oxy release in rats (Kiss et al., 2010). Teltsh et al. (2012) found an association between one variant in the 5′-as well as 3′promoter region of oxy and schizophrenia in a large clan of Arab-Israeli individuals. The first study conducted on men with the “simple form of schizophrenia” and subjected to intravenous or intranasal oxy administration revealed an improvement of depression and negative symptoms (Bakharev et al., 1986). When oxy was administred intranasally to healthy, polydipsic, and nonpolydipsic patients with schizophrenia, who were asked to rate the presence and intensity of various facial emotions, Goldman et al. (2011) demonstrated that emotion recognition was improved in polydipsic patients but not in non polydipsic patients. Moreover, it has been demonstrated that oxy improves cognition in schizophrenia, as suggested by the findings obtained by Feifel et al. (2012) demonstrating the ability oxy treatment to improve verbal memory learning tasks.

In the last years it has been evaluated the effect of the chronic exposure to a combination of intranasal oxy and atypical antipsychotic in schizophrenic patients; the results obtained demonstrated that oxy improved negative symptoms when administered twice per day in a range between 3 and 6 weeks (Feifel et al., 2010; Modabbernia et al., 2013; Gibson et al., 2014). On the other hand, the group of Davis et al. (2014) did not report any improvement of negative symptoms after a single dose of intranasal oxy given twice a week before a session of social skills training. It is worth to emphasize that these clinical trials, which suggest the possible beneficial role of intranasal administration of oxy, are in contrast with preclinical studies performed on laboratory rodents. In fact a number of recent findings suggest that the chronic treatment with oxy not only produce no benefit, but may further worsen social interaction in rodents (Bales et al., 2013; Rault et al., 2013; Huang et al., 2014). Finally in a very recent study (Shin et al., 2015), in which the authors evaluated the effect of a single dose of intranasal oxy on brain activity in patients with schizophrenia, oxy was able to modulate the neuronal response to different facial emotions. Although more and larger studies are necessary to determine the impact and the generalizability of the findings described above, overall, oxy shows great promise as being a possible and effective treatment for patients with schizophrenia.

As far as mood disorders, a large body of studies have examined the implication of the oxytocinergic system in both major depressive disorder (MDD) and bipolar disorder (BD) and the findings suggest that oxy-related physiological functions change in patients with mood disorders.

In particular, in a post mortem study it has been found that both the number of oxy-producing neurons and oxy mRNA levels within the PVN were increased in patients with MDD (Purba et al., 1996; Meynen et al., 2007). Moreover, the CSF levels of hNPII were higher in bipolar depressed patients compared to healthy controls (Legros et al., 1983; Linkowski et al., 1984), although several studies reported no differences in the CSF hNPII (Linkowski et al., 1984), plasma hNPII (Scantamburlo et al., 2005), CSF oxy (Pitts et al., 1995), or plasma oxy levels (van Londen et al., 1997; Sasayama et al., 2012) in patient with MDD.

In contrast to these findings, Frasch et al. (1995) demonstrated that nocturnal plasma oxy levels were decreased in patient with MDD compared with age-matched controls and that these differences were more pronounced in old than in young patients. On the same line, it has been reported a decrease in serum oxy concentrations in patients with MDD or BD, compared with healthy controls (Ozsoy et al., 2009).

On the contrary, results obtained from a small study (11 MDD, 19 healthy control patients) reported that oxy plasma concentrations increase during the night in patients with MDD compared to healthy controls. From the observations reported above, it is clear that the results are conflicting and recent studies, performed to elucidate factors that may contribute to these differences across studies, revealed that the dysregulation of oxytocinergic system in MDD may be due to the kind of task used and that any measurement of oxy levels at different time point may lead to conflicting results. For example Scantamburlo et al. (2007) found that oxy levels negatively correlated with Hamilton Depression Rating Scales scores (HAM-D) and anxiety scores on the State-Trait Anxiety Inventory while a positive correlation of oxy plasma levels was found with the Temperament and Character Inventory in outpatients with MDD (Bell et al., 2006). Finally, (Cyranowski et al., 2008) demonstrated that women with MDD showed more variability in oxy release during the different sessions of the task as compared to healthy control group.

The association between the oxyr genetic variations and mood disorders has been studied. In particular adult patients with MDD showed differences in two SNPs within the oxyr gene (Costa et al., 2009) already associated with ASD and a recent study reported for the first time an association between early life stress and symptoms and the oxyr rs139832701 variant (Myers et al., 2014). Multiple studies investigated the response of oxy levels to electroconvulsive therapy (ECT), a standard psychiatric treatment used as a last line of intervention for MDD. All together these studies suggest that the release of oxy and its related carrier protein hNPII could be implicated in the beneficial effect of ECT (Whalley et al., 1982, 1987; Smith et al., 1990, 1994; Scott et al., 1986, 1989, 1991). Although one case report (Scantamburlo et al., 2011) recording the effects of oxy on MDD revealed that 3 weeks intranasal oxy treatment lead to a decrease in depressive and anxiety-related symptoms a larger research in this context is necessary.

Several lines of evidence have demonstrated that oxy is an important regulator of anxiety related to physiological stress response; the anxiolytic effect of oxy occurs mainly at the level of PVN and amygdala (Neumann et al., 2000; Bale et al., 2001; Blume et al., 2008; Viviani et al., 2011; Knobloch et al., 2012). Interestingly, a very recent preclinical study revealed that administration od oxy in the central nucleus of the amygdala induces an anxiolytic effects and that this positive effect is abolished by the local pharmacological blockade of oxyrs in this area (László et al., 2015).

In mothers oxy levels positively correlate with sociality, calm, and tolerance (Nissen et al., 1998) and with a less probability of the occurrence of anxiety and stress disorders (Altemus, 1995). Interestingly, during pregnancy and lactation, when oxy levels increase, the mother is protected form anxiety disorders and breastfeeding induces a reduction of stress hormones, as compared with women who bottle-feed their infants (Altemus, 1995). Moreover, women with panic disorder reported a reduction of anxiety symptoms during lactation (Klein et al., 1994–1995) and the anxiolytic action of oxy is enhanced in the presence of circulating oestrogens (McCarthy et al., 1996). Interestingly, intranasal oxy administration reduces amygdala activation, a brain area often dysfunctional in depression and implicated in the biological response to fear (Kirsch et al., 2005). More than one study reported plasma oxy levels in patients with Generalized Social Anxiety Disorder (GSAD). Although social anxiety disorders were associated with an increase of oxy levels, the study from Hoge et al. (2008) reported no significant differences between GSAD and control patients. In another study (Hoge et al., 2012), patients with GSAD had similar plasma oxy levels as compared to controls, but oxy levels were decreased after completing a trust game with a partner. Finally, intranasal oxy administration to patients with GSAD has improved their performance during a speech in front of a group of individuals, and improved mental representations of self (Guastella et al., 2009). In a similar study, a decrease in anxiety symptoms and negative self-appraisals has been found when oxy was administered before an impromptu speech task (Alvares et al., 2012). Finally, a very recent study demonstrated that an oxy analog namely LOT-1, able to penetrate better the BBB and with long-lasting effect significantly improved anxiety-like behavior and social avoidance in CD157 knockout mouse (a model of non-motor symptoms of Parkinson's disease). Interestingly, LOT-1 had a greater effect on rescue 24 h from its injection, suggesting that this compound have a longer half-life and a long-lasting effect as compared to oxy (Mizuno et al., 2015).

Among anxiety disorder, OCD is characterized by obsessions and/or compulsions or a combination of such obsessions and compulsions. Oxyrs have been identified in some brain areas, which were linked to the pathophysiology of OCD (Rapoport and Wise, 1988; Modell et al., 1989).

Central oxy administration induces grooming behavior in animals (Witt et al., 1990; Van Erp et al., 1993; Drago et al., 1999), which is considered a model of compulsion as cleaning behavior is a typical symptom displayed by OCD patients (Holzer et al., 1994; Leckman et al., 1994–1995; McDougle et al., 1999). However, the most consistent data regarding the connection between oxytocinergic system and OCD came from the evidence that women display an increased risk to develop a subtype of OCD during pregnancy and the post-partum period, both conditions characterized by elevated levels of oxy (Jenike, 1990; Neziroglu et al., 1992; Sichel et al., 1993). Moreover, adults with OCD showed an increase in oxy CSF levels as well as people affected by the Tourette's syndrome, (Leckman et al., 1994) a neuropsychiatric disorder characterized by multiple physical (motor) and vocal (phonic) tics. However, controversial results have been also published (Altemus et al., 1999) so that more experimental data are necessary to definitely asses the potentiality of oxy (or oxyr antagonists) as anti-obsessional treatment.

### Oxytocin and drug addiction

Studies focused on the role of oxy on drug effects and addiction began to emerge in the 1980 (Sarnyai and Kovács, 1994). Since then, theories on the possible involvement of oxy in addiction have begun to receive more and more attention. Interestingly, oxy is released in response to acute 3,4-methylenedioxy-methamphetamine (MDMA, “ecstasy”) and methamphetamine administration in both humans (Wolff et al., 2006; Dumont et al., 2009) and animals (Thompson et al., 2007; Broadbear et al., 2011).

In animal studies, oxy was able impede tolerance development to morphine (Kovács and Telegdy, 1987) and dampen the severity of the effects induced by morphine withdrawal (reviewed by Kovács et al., 1998); moreover, oxy administration in rats, leads to a reduction of self-administration of heroin (Kovács and Van Ree, 1985; Kovács et al., 1985; Ibragimov et al., 1987). Oxy attenuates also the hyperactivity due to cocaine use and inhibits the development of tolerance to this drug (Kovàcs et al., 1990; Sarnyai et al., 1992a,b). As far as alcohol, oxy prevents the development of tolerance to ethanol in laboratory mice (Szabó et al., 1989) although it has been demonstrated that acute alcohol administration inhibits oxy secretions while its chronic use is able to stimulate it (Marchesi et al., 1997). Moreover, it has been suggested that oxy might exerts a role in the cognitive dysfunctions observed in alcoholics (Holden et al., 1988; Marchesi et al., 1997). All drugs of abuse increase dopamine release within the mesolimbic system (Pierce and Kumaresan, 2006) and Young and collaborators (Young et al., 2008, 2011) demonstrated the existence of an interaction between oxy and dopaminergic system in both social and drug reward in prairie voles. In particular, the authors showed that methamphetamine was able to reduce pair bonding and pair bonding was able to reduce the rewarding properties of methamphetamine in prairie voles. Interestingly, the interaction occurring within the nucleus accumbens between the mesolimbic dopaminergic and oxytocinergic system was of crucial relevance in this behavior: drugs in prairie voles could reduce dopamine release after social interaction, and social interaction could reduce the reward from drugs. Moreover, it is well known that oxy acts on oxyrs in the medial preoptic area resulting in an increase of dopamine release from VTA neurons (Champagne et al., 2004; Shahrokh et al., 2010) and this effect is reduced by the administration of an oxyr antagonist in the VTA (Shahrokh et al., 2010); moreover, oxy injections in the VTA leads to an increase of dopamine signal in the NAc (Shahrokh et al., 2010). It would be very interesting to evaluate if this interaction does also exists in humans. Interestingly, Love et al. (2012) have recently shown that oxy gene polymorphisms in humans influence dopaminergic function in a gender-specific manner.

## Conclusions

A combination between genetic, epigenetic, and environmental factors contributes to originate several neuropsychiatric disorders including ASD, eating disorders, anxiety, depression, and several more. Most of the therapies in use are based on drugs with often limited efficacy and whose mechanisms of action are still not well characterized. Together with classical neurotransmitters, neuropeptidergic signaling is receiving increasing attention since neuropeptides are signaling molecule involved in a wide range of brain functions including stress, reward, food intake, metabolism, reproduction, social behaviors, learning, and memory. Although the evidence reviewed here suggests the implication of the oxytocinergic system in several behaviors (Table 1) associated with neuropsychiatric dysfunctions, a number of critical questions still remain to be addressed, in view of a possible drug development along this line. In fact larger, randomized and more controlled trials are needed to better understand the role played by the oxytocinergic system in the pathophysiology of the disorders and the possible use of drugs affecting this system (agonist/antagonist) as novel therapeutic agents in this context.

**Table 1:** **Effects of oxytocin on several behavior in different species**.

	**Species**				
**Behavior**	**Rat**	**Mouse**	**Parairie voles**	**Human**	**References**
Maternal behavior	↑	↑	?	↑	Russell et al., 2003; Leng et al., 2005; Ross and Young, 2009; Bosch and Neumann, 2012
Affiliative behavior	↑	↑	↑	?	Dantzer et al., 1987; Williams et al., 1994; Insel and Hulihan, 1995
Sexual behavior	↑	↔	↓male ↑ or ↓ female	↑	Carmichael et al., 1987; Carter, 1992; Anderson-Hunt and Dennerstein, 1994, 1995; Melis et al., 2007; Baskerville et al., 2009; Gil et al., 2011; Lazzari et al., 2013
Nociception	↓	↓	?	↓	Yang, 1976; Lundeberg et al., 1993; Xu and Wiesenfeld-Hallin, 1994; Condés-Lara et al., 2005; Reeta et al., 2006; Gu and Yu, 2007; Yang et al., 2007a,b; Han and Yu, 2009; Mazzuca et al., 2011
Social behavior	?	prevents deficits in social behavior and learning ability	?	↑ retention of social information and social communication ↓ repetitive behavior	Ferguson et al., 2002; Hollander et al., 2003, 2007; Kosaka et al., 2012; Tachibana et al., 2013; Teng et al., 2013; Meziane et al., 2014; Peñagarikano et al., 2015
Feeding	↓	↓	?	↓	Arletti et al., 1989, 1990; Olson et al., 1991; Kublaoui et al., 2008; Takayanagi et al., 2008; Maejima et al., 2009, 2011; Deblon et al., 2011; Dombret et al., 2012; Morton et al., 2012; Ott et al., 2013; Altirriba et al., 2014; Blevins et al., 2015; Iwasaki et al., 2015
Depressive and anxiety related behavior	↓	↑	?	↓	Bakharev et al., 1986; Altemus, 1995; Neumann et al., 2000; Bale et al., 2001; Blume et al., 2008; Viero et al., 2010; Dabrowska et al., 2011; Scantamburlo et al., 2011; Viviani et al., 2011; Knobloch et al., 2012; László et al., 2015; Mizuno et al., 2015
Grooming	↑	↑	↑	↑	Witt et al., 1990; Van Erp et al., 1993; Holzer et al., 1994; Drago et al., 1999;
Tolerance to opiates	↓	↓	?	?	Kovàcs et al., 1990; Sarnyai et al., 1992a,b; Kovács et al., 1998;
Tolerance to ethanol	?	↓	?	?	Szabó et al., 1989

*The effects of oxytocin on behaviors are indicated as follows: ↑, increase; ↔, no effect; ↓, decrease; ?, unknown (for details, see the indicated references and additional references in the text)*.

## Funding

We thank MIUR for the grant RBFR12DELS, which covers the post-doctoral fellowship of Dr. AR and of Dr. MM.

### Conflict of interest statement

The authors declare that the research was conducted in the absence of any commercial or financial relationships that could be construed as a potential conflict of interest.
